# Transplantation of a beating heart: A first in man

**DOI:** 10.1016/j.lanwpc.2022.100449

**Published:** 2022-04-12

**Authors:** Shengli Yin, Jian Rong, Yinghua Chen, Lu Cao, Yunqi Liu, Shaoyan Mo, Hanzhao Li, Nan Jiang, Han Shi, Tielong Wang, Yongxu Shi, Yanling Zhu, Wei Xiong, Yili Chen, Guixing Xu, Xiaoxiang Chen, Xiaojun Chen, Meixian Yin, Fengqiu Gong, Wenqi Huang, Yugang Dong, Nashan Björn, Tullius Stefan, Zhiyong Guo, Xiaoshun He

**Affiliations:** a Department of Cardiac Surgery, The First Affiliated Hospital, Sun Yat-sen University, Guangzhou 510080, China; b Department of Extracorporeal Circulation, The First Affiliated Hospital, Sun Yat-sen University, Guangzhou 510080, China; c Organ Transplant Centre, The First Affiliated Hospital, Sun Yat-sen University, NO.58 Zhongshan Er Road, Guangzhou 510080, China; d Guangdong Provincial International Cooperation Base of Science and Technology (Organ Transplantation), Guangzhou 510080, China; e Guangdong Provincial Key Laboratory of Organ Donation and Transplant Immunology, Guangzhou 510080, China; f Department of Anesthesiology, The First Affiliated Hospital, Sun Yat-sen University, Guangzhou 510080, China; g Department of Cardiology, The First Affiliated Hospital, Sun Yat-sen University, Guangzhou 510080, China; h Department of Neurosurgery, The First Affiliated Hospital, Sun Yat-sen University, Guangzhou 510080, China; i Operating Room and Anesthesia Centre, The First Affiliated Hospital, Sun Yat-sen University, Guangzhou 510080, China; j Organ Transplantation Centre, The First Affiliated Hospital of the University of Science and Technology of China, Hefei 230001, China; k Division of Transplant Surgery, Brigham and Women's Hospital, Harvard Medical School, Boston, MA, USA

## Abstract

**Background:**

In the current practice, graft ischaemia and reperfusion injury (IRI) is considered an inevitable component in organ transplantation, contributes to compromised organ quality, inferior graft survival and limitations in organ availability. Among all the donor organs, the heart is most vulnerable to IRI and the tolerated ischaemic time is the shortest.

**Methods:**

By combining adapted surgical techniques and normothermic machine perfusion (NMP), we performed the first case of ischaemia-free beating heart transplantation (IFBHT) in man. The donor heart was procured after an *in situ* NMP circuit was established, then underwent *ex situ* NMP and implanted under NMP support. The post-transplant graft function was monitored.

**Findings:**

The donor heart was procured, preserved, and implanted under a continuously perfused, normothermic, oxygenated, beating state. During *ex situ* NMP, the donor heart beat with sinus rhythm and adequate ventricular contraction, consumed oxygen and lactate, suggesting a good cardiac function. The dynamic electrocardiogram demonstrated an absence of ischaemic injury of the donor heart during the entire procedure. The echocardiogram showed an immediate graft function with a left ventricle ejection fraction (LVEF) of 70%. The patient was discharged on post-transplantation day 20 and was followed up for 8 months with normal cardiac function and life.

**Interpretation:**

This study shows the feasibility of IFBHT procedure, which might be able to completely avoid graft IRI, has thus the potential to improve transplant outcome while increasing organ utilization.

**Funding:**

This study was funded by National Natural Science Foundation of China, Guangdong Provincial Key Laboratory Construction Projection on Organ Donation and Transplant Immunology, and Guangdong Provincial International Cooperation Base of Science and Technology.

**Research in context**

**Evidence before the study**

We searched PubMed using the terms ‘machine perfusion’, ‘*ex vivo* perfusion’, ‘cardiac transplantation’ or ‘heart transplantation’ for relevant articles in any language published until December 01, 2021. We searched for clinical studies on heart transplantation using machine perfusion as a preservation method. Previous studies have documented the safety and efficacy of *ex situ* normothermic machine perfusion and hypothermic oxygenated perfusion (also called nonischaemic heart preservation) for heart transplantation. However, the donor heart still suffers ischaemia during procurement and implantation. Ischaemia-free organ transplantation (IFOT) is an entirely new approach during which the organ is procured, preserved and implanted with continuous blood supply. Our group has shown the feasibility of this approach in liver and kidney transplantation.

**Added value of the study**

This is the first report of ischaemia-free beating heart transplantation (IFBHT), confirming that this approach is feasible and safe. Considering that the donor heart is the most susceptible organ to ischaemia reperfusion injury, IFBHT offers an opportunity to improve transplant outcome and increase organ availability in heart transplantation.

**Implications of all the available evidence**

The concept of IFOT is feasible in liver, kidney and heart transplantation. By avoiding graft ischemia, IFOT is a promising approach to overpass organ shortage and post-transplant complications. Future studies should show “hard” clinical advantage, optimize the technology, and provide a positive cost-effectiveness analysis.

Alt-text: Unlabelled box

## Introduction

Heart transplantation is the gold standard treatment for patients with end-stage heart failure.^1^ However, the success of heart transplantation continues to be limited by a paucity of donor organs and suboptimal transplant outcome.^2^, ^3^, ^4^ In the current practice, the majority of donor organs are procured after being flushed with a cold preservation solution and then undergo static cold storage. Finally, all the organs are implanted under hypothermic and ischaemic conditions. A degree of graft ischaemia and reperfusion injury (IRI) is thus considered an inherent and unavoidable component of organ transplantation.^5^

Among all the donated organs, the donor heart is the most vulnerable to IRI, which largely limits the selection of donor hearts. For instance, donor hearts with an ischaemic time over 6 h or age older than 60 years are frequently discarded.^6^ Importantly, IRI contributes to early graft failure, the most frequent cause of death within the first month post-heart transplantation.^6^^,^^7^ IRI also contributes to allograft rejection and cardiac allograft vasculopathy (CAV), which are the major reasons for long-term graft loss and patient death following heart transplantation.^6^ Therefore, reduction of graft IRI is undoubtedly the most important single technical factor in the determinant of success of heart transplantation.

To ameliorate donor heart IRI, efforts have been made in developing novel protection and preservation methods.^8^ Among these efforts, the normothermic machine perfusion (NMP) technique has been applied clinically.^9^ This approach allows preservation and transportation of a beating, normothermic, oxygenated, and perfused heart and has the capacity to ameliorate the consequences of IRI. Nevertheless, this approach still entails a period of ischaemia during the procurement of the heart after cold cardioplegic arrest and during donor heart implantation under hypothermic and hypoxic conditions. In this scenario, the donor heart might even suffer a “double-hit” of IRI, limiting the potential of NMP.

We have taken an entirely novel approach avoiding ischaemia during heart transplantation and have developed an ischaemia-free beating heart transplantation (IFBHT) procedure. This approach is based on extensive pre-clinical work in large animals. Herein we present the first IFBHT in man.

## Methods

### Recipient and donor assessment

A 67-year-old male patient, presented with chest tightness and shortness of breath for four months. The echocardiogram (ECHO) study showed enlargement of the left and right atrium, and the left ventricle, with a left ventricle ejection fraction (LVEF) of 27%, and an estimated pulmonary artery systolic pressure (PASP) of 56 mmHg. The contrast enhanced cardiac magnetic resonance (MR) study showed enlargement of the heart with extensive myocardial fibrosis; based on morphological changes together with a LVEF of 16%, the patient was diagnosed with a non-ischaemic dilated cardiomyopathy. Therefore, the patient was listed for a heart transplant. Preoperatively, the patient needed to undergo a surgical placement of an intra-aortic balloon pump (IABP) through the femoral artery. The patient was also receiving non-invasive ventilation for respiratory support.

On June 26th, 2021, a donor heart from our hospital was allocated to this patient through the China Organ Transplant Response System (COTRS). The donor was a 29-year-old man who died of a head trauma. Brain death was confirmed after intensive care treatment for 10 days. There was no history of cardiopulmonary resuscitation prior to organ donation. The pre-procurement creatine kinase MB (CK-MB) isoenzyme, and cardiac troponin I (cTnI) were 56 unit per litre and 0.012 ng per mililitre. The electrocardiogram (ECG) findings were within normal range (appendix p 3). An ECHO could not be done due to the presence of pneumomediastinum.

### Intraoperative donor management

During organ procurement, the donor was managed according to international standards.^10^ Additional monitoring included a transesophageal echocardiography (TEE), revealing a PASP of 49 mmHg and a LVEF of 70%. As per standard cardiopulmonary bypass (CPB) procedure, heparin was administered before aortic cannulation. The main goal of ventilatory management should minimize any increase in peripheral vascular resistance (PVR) and prevent its detrimental effects on the right ventricle. Succinylated gelatin injection was used for volume replacement to maintain stable hemodynamic stabilization.

### *Ex situ* heart NMP

**Figure 1:**
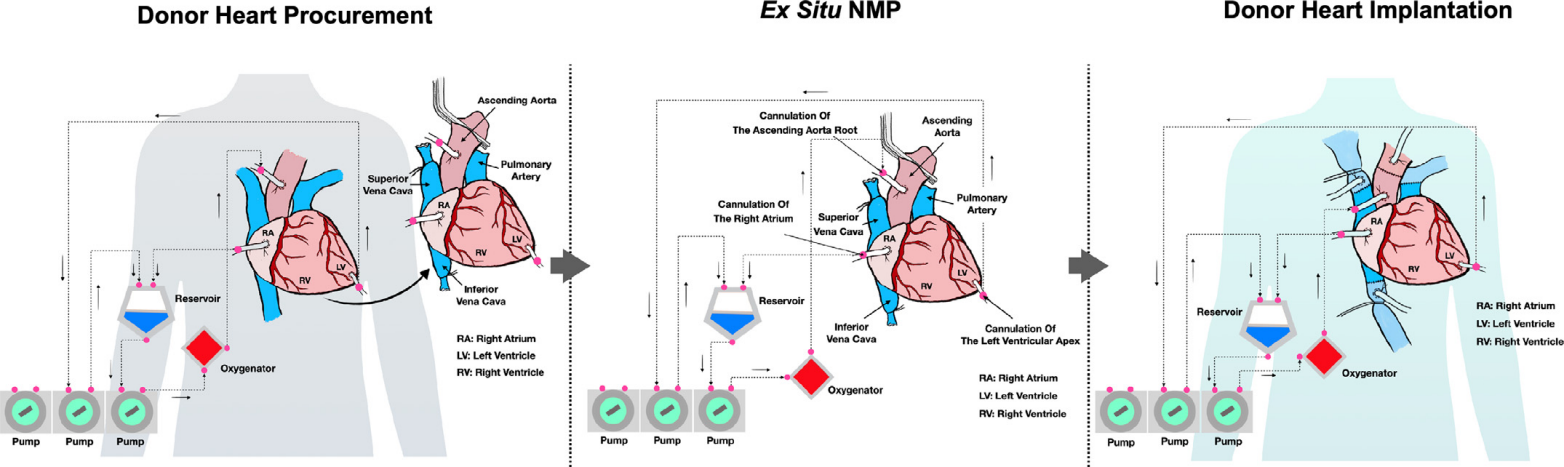
**The ischaemia-free beating heart transplantation procedure.** The aortic root (for perfusion), right atrium and the left ventricular apex (for drainage) are cannulated and connected to the heart-lung machine. The donor heart is procured after the *in situ* normothermic machine perfusion (NMP) circuit is established, then moved to the organ reservoir and underwent continuous *ex situ* NMP*.* The donor heart is implanted under *in situ* NMP. The donor left atrium and aorta are firstly anastomosed to the recipient counterparts. Subsequently, the donor heart is reperfused with the recipient's blood. Finally, the donor inferior vena cava, superior vena cava, and pulmonary artery are anastomosed end-to-end to the recipient counterparts.

The heart NMP was conducted using a heart–lung machine (S5, LivaNova, Germany). The main components include an automatic pressure and flow-controlled perfusion system, a membrane oxygenator (Affinity Pixie, Medtronic Inc, Minneapolis, MN, USA), a leucocyte filter, an arterial filter, a heater-cooler unit, an autonomous power unit, and a programmable sequencer (Figure 1). Around 600 ml of donor blood was collected and passed through a leucocyte filter in preparation of the machine perfusion perfusate. The perfusion system was primed with 300 ml of donor blood, and 100 ml of priming solution containing buffered electrolytes, mannitol, vitamins, and steroids (Table 1).

**Table 1:** The components of the perfusate solution.

Components	
Priming Solution (per 500 ml)	
Mannitol	12.5 g
Sodium Chloride	2.63 g
Potassium Chloride	185 mg
Sodium Gluconate	251 mg
Sodium Acetate	184 mg
Magnesium Chloride	150 mg
Sodium Bicarbonate	20 mEq
Mythylprednisolone	200 mg
Multivitamins	5 ml
Heparin	2500 IU
Ceftriaxone Sodium and Tazobactam Sodium	1.5 g
25% Albumin	100 ml
Heart Maintenance Solution (per 500 ml)	
Calcium Chloride	1.2 g
Magnesium Sulfate	0.2 g
Potassium Chloride	0.01 g
Sodium Chloride	0.825 g
Nicorandil	12 mg
Amino Acids	3%
Catecholamine Maintenance Solution	
Epinephrine 1 mg in 50 ml of 0.9% Normal Saline	0.6–18 ug per min

During *ex situ* NMP, the perfusion pressure, flow, temperature and contractility of ventricles were monitored. The arterial and venous perfusate samples were collected and analysed with a handheld lactate analyser (i-Stat, Abbott Diagnostics, East Windsor, NJ, USA). The heart rate and rhythm were monitored by ECG and visual assessment.

The composite definition of transplantability included (1) the perfusate pH value higher than 7.3 without continuous use of bicarbonate; (2) evidence of lactate clearance, including a downward trend of the lactate level in the perfusate or presence of arteriovenous lactate gradient with a final lactate level lower than 5.0 mmol per litre; (3) a coronary vascular flow of greater than 500 ml per min under a perfusion pressure of 60–100 mmHg; (4) heart beating with sinus rhythm rate of around 60–100 beat per min; (5) adequate right ventricular contraction without obvious evidence of wall motion abnormality. The donor heart was only accepted when it met all the above criteria.

### Intraoperative recipient management

A pulmonary artery catheter (PAC) was placed under ultrasound guidance for PVR or trans-pulmonary gradient and pulmonary artery pressure measurement at the start of surgery. The central venous pressure was then monitored continuously, and blood gas analysis was performed intermittently to maintain a stable inner milieu. A TEE probe was placed after the induction of anesthesia, and a comprehensive examination was performed for early detection of hemodynamic instability. During operation, the anesthetic depth was titrated to maintain it at the E2-D1 level on Narcotrend monitoring (MonitorTechnik, Bad Bramstedt, Germany) with hemodynamic stability.

Post-CPB anesthetic management required close monitoring of PVR and pulmonary pressures with a PAC and a comprehensive TEE evaluation. Initial TEE focused on thorough de-airing of the cardiac chambers and on graft function.

### Post-transplant patient care

The post-transplant care was done according to our standard protocol. The patient received an induction therapy with Basiliximab (20 mg) intraoperatively and on day 4. Tacrolimus (initial dose of 1.5 mg q12h) and mycophenolate (initial dose of 0.36 g q12h) were used for maintenance therapy with a target trough FK506 level of 8–10 ng/ml. Sulperazone, Vancomycin and Micafungin Sodium were used for prophylaxis therapy.

The study protocol (2021-02) was approved by the Ethical Committee of The First Affiliated Hospital, Sun Yat-sen University.

### Role of the funding source

Funding did not influence study design, data collection, data analysis, data interpretation, or writing of the manuscript. The corresponding authors had full access to all data at any time and take final responsibility for the submission.

## Results

### Ischaemia-free donor heart procurement

**Figure 2:**
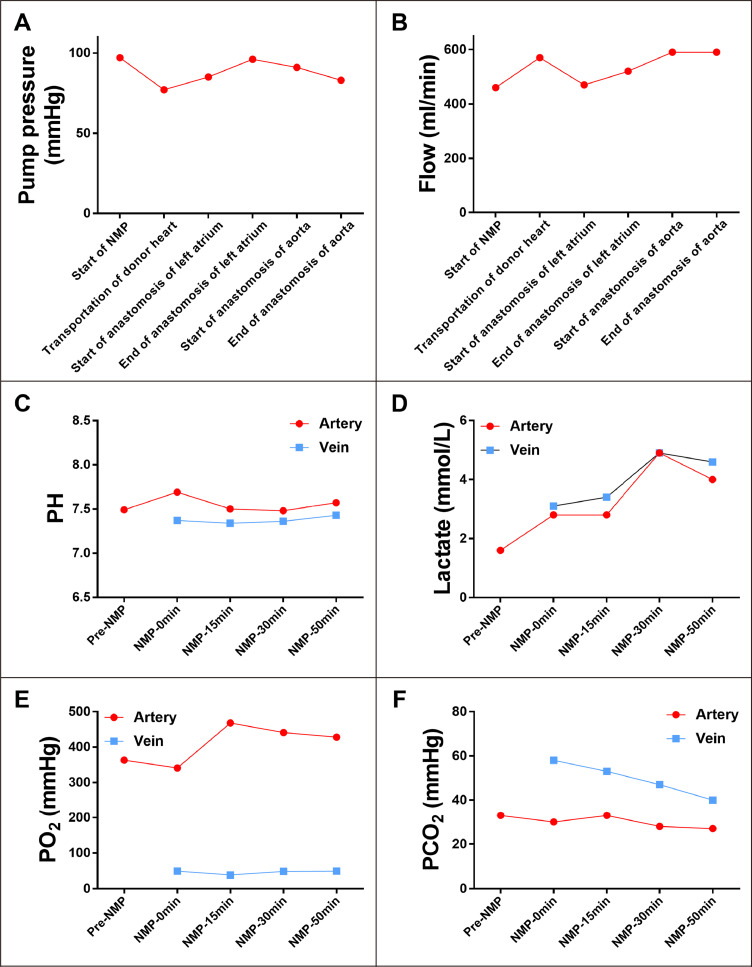
**The normothermic machine perfusion (NMP) of donor heart.** Panel A and B show the perfusion pressure (mm Hg) and flow (ml per min) of the coronary artery. Panel C and D show the pH value and lactate level (mmol per litre) of the arterial and venous perfusate. Panel E and D show the oxygen (PO_2_, mmHg) and carbon dioxide (PCO_2_, mmHg) tension of the arterial and venous perfusate.

Figure 1 and Video 1 show the details of the IFBHT technique. The donor heart was procured in a continuous beating state. A median sternotomy was performed using a sternal saw. The aorta, superior vena cava, and inferior vena cava were dissected and umbilical tapes were positioned circumferentially. The aortic root and right atrium were sutured in a purse-string manner. After the donor was fully heparinized, the aortic root (for perfusion), right atrium and left ventricular apex (for drainage) were cannulated and connected to the heart-lung machine. The superior and inferior vena caval veins were clamped. The distal ascending aorta was cross-clamped and *in situ* retrograde coronary perfusion was started through the proximal ascending aorta directly from the heart-lung machine. The pulmonary artery and left atrium were cut open to vent the heart and maintain it in a decongested state. The pump flow was adjusted to maintain the mean aortic pressure between 60 and 100 mm Hg (Figure 2A). Both the superior vena cava and inferior vena cava were ligated and divided. Subsequently, the heart was harvested and moved to the organ reservoir. The donor heart procurement time was 15 min. The family members of the donor provided informed consent of donating the heart, liver and two kidneys. Therefore, the liver and kidneys were also procured using a standard fast cold flush method after the ascending aorta was clamped.

### *Ex situ* donor heart NMP

The heart graft was perfused with an oxygenated blood-based solution (Table 1) in a normothermic state *ex situ*. A maintenance solution containing isotonic electrolytes, aminoacids, and nicorandil was infused at a rate of 0–30 ml per hour. Nicorandil and adrenaline, as well as an adjustable circuit pump flow, were used to assure a coronary vascular flow of greater than 500 ml per min (Figure 2B) and keep the sinus rhythm rate of around 60–100 beat per min. The venous PH values were within normal range (Figure 2C). The arterial and venous lactate were 2.8–4.9 and 3.1–4.9 mmol per litre, respectively, with a downward trend after 30 min of NMP (Figure 2D). The arteriovenous gradient showed a substantial consumption of oxygen and production of carbon dioxide (Figure 2E and F). In addition, the heart exhibited adequate right ventricular contraction without obvious evidence of wall motion abnormality, although the visual assessment of left ventricular contraction is inherently limited in a resting mode configuration. These parameters collectively suggest a good graft viability. During *ex situ* NMP, the integrity of the superior vena cava, inferior vena cava, ascending aorta, pulmonary artery, atria and ventricles was confirmed. The left atrial cuff was created by incisions between each of the four pulmonary veins to create a smooth continuous edge. The mitral valve was working normally. The total heart NMP duration was 65 min.

### Ischaemia-free heart implantation

The donor and recipient operations were performed in two nearby operation rooms in our hospital. The CPB and excision of the diseased heart of the recipient were conducted using a standard method. The beating donor heart was placed *in situ* in the recipient without discontinuation of normothermic perfusion. The donor left atria cuff was anastomosed end-to-end to the recipient left atria with running sutures using 3-0 Prolene. The right superior pulmonary vein vent was inserted into the left ventricle through the mitral valve. The donor and recipient ascending aortas were anastomosed with running suture using 4-0 Prolene. 400 ml washed red blood cells were used to rinse the donor heart. In the meanwhile, air-evacuating maneuvers were performed in the aorta and left ventricle. The recipient received 500 mg Methylprednisone prior to the discontinuing the NMP of the donor heart. The cross clamp was gently released, and the recipient blood perfusion of the donor heart was established.

The donor inferior vena cava, superior vena cava, and pulmonary artery were anastomosed end-to-end to the recipient counterparts. Hemodynamics and blood gas analysis were stable, while vasoactive drugs were reduced and a sufficient urine volume was observed. CPB (132 min) was thus weaned off and the use of IABP was resumed. Drainage tubes were placed in the pericardium and mediastinum.

On successful transition from CPB and removal of venous and aortic cannulae, protamine was given to reverse the heparin. Immediate evaluation of post-bypass coagulopathy was performed to ensure adequate reversal. The heart exhibited satisfactory contractility and pulmonary artery pressure after weaning from CBP. The heart rate, rhythm and blood pressure were stable. Video 2 shows the intraoperative TEE monitoring with synchronous dynamic ECG monitoring. The ECG showed no abnormality of the ST segments. The intraoperative TEE showed an immediate graft function with a LVEF of 70% (Video 2). Lactated Ringer's solution (1500 ml), normal saline (600 ml) and fresh frozen plasma (200 ml), washed red blood cells (460 ml) plus platelets (1 unit) were infused during the operation.

The recipient operation time was 308 min, and the patient was transferred to the intensive care unit (ICU) for close monitoring.

### Post-transplant follow up

The recipient received a routine immunosuppression and antibiotics therapy in our center. The patient was extubated by day 2. The use of vasoactive drugs and IABP was discontinued on day 3. Although the levels of myocardial injury markers increased post-transplantation (appendix p 2), daily ECG showed no obvious evidence of myocardial ischaemia (appendix p 3). Daily ECHO demonstrated normal cardiac function with a LVEF greater than 75%; PASP dropped to 16 mm Hg post-transplantation. The patient had an acute kidney injury which recovered without the requirement for hemodialysis. Acute rejection and vascular complications did not occur. The patient was discharged on post-operative day 20. A routine chest CT scan showed multiple cavities in the left and right upper lungs on day 34, suggesting a fungal infection. The patient had no fever, cough and shortness of breath. After antifungal therapy, the cavities became smaller. Until this report written, the patient has been followed up for 8 months with normal graft function as assessed by ECHO with LVEFs greater than 60%.

## Discussion

Since the first successful human heart transplant in 1967,^1^ the majority of donor hearts continue to be procured following cold cardioplegic arrest, subsequently preserved, transported, and implanted in a hypothermic and hypoxic condition.^11^ Graft IRI is an unavoidable event in the current practice. The availability, viability and quality of donor hearts can therefore be compromised with the increased likelihood of poorer outcomes post-transplantation. To the best of our knowledge, this case represents the first ischaemia-free, normothermic, oxygenated, beating heart transplantation in human. Therefore, the IFBHT technique has a potential to increase graft availability and improve transplant outcomes.

During NMP, we confirmed the excellent quality of the cardiac graft by measuring perfusion pressure and flow; we also observed a downward trend of the lactate profile after NMP, as well as normal sinus rhythm and myocardial contractility. Importantly, the intraoperative TEE monitoring and visual inspection indicated continuous excellent cardiac function during the whole procedure. Therefore, the graft viability and function are well preserved during IFBHT. The levels of troponins and natriuretic peptides still increased post-transplantation, although no obvious ECG change indicated the presence of myocardial ischaemia. It is still unknown whether IFBHT can reduce the levels of these markers when compared to the conventional technique. The gold standard to assess graft IRI is pathological assessment of biopsy samples. For the safety of the patient, we did not conduct a biopsy in this first case. We are developing a protocol to conduct biopsies pre-procurement, at the end of NMP and after reperfusion during IFBHT to assess graft IRI.

There are at least two *ex situ* heart machine perfusion strategies. The organ care system (OCS) is to perfuse the heart in a normothermic, oxygenated, beating state.^9^^,^^12^ In contrast, the XVIVO system perfuses the heart under hypothermic, oxygenated, cardioplegic conditions.^13^ A direct comparison of these techniques has not been performed. Of note, continuous machine perfusion has thus far only been used during heart preservation. The cardiac allograft will therefore still undergo a period of ischaemia during procurement and implantation. In contrast, the IFBHT technique has been designed to completely avoid graft ischaemia during the entire procedure of heart procurement, preservation and implantation. There is no commercially available portable heart machine perfusion device in our country at this time, which limits the procurement of distant donor hearts. However, the concept of ischaemia-free (beating or non-beating) heart transplantation can be applied with the use of both OCS and XVIVO systems, after modification of the techniques as shown in our case. In addition, because the cannulation of the donor heart used in IFBHT is quite similar with that in conventional technique, the multi-organ procurement can be performed using a standard cold flush technique without introduction of extra ischaemia. Ideally, all the donor organs should be simultaneously procured using an ischaemia-free technique. However, the practice is currently limited by the logistics conditions.

There are certain limitations concerning IFBHT that require further innovation. Firstly, this report only showed the feasibility of IFBHT. A well-designed trial to demonstrate both short-term and long-term benefits of our approach is on the way. Secondly, the heart was from a standard criteria donation after neurological determination of death (DNDD) donor in this case. The extended criteria donor hearts probably benefit the most from the novel procedure. The hearts from donation after circulatory determination of death (DCDD) donors might also benefit from IFBHT after regional normothermic perfusion.^14^ Thirdly, unlike in *ex situ* liver NMP,^15^ the criteria of transplantability of the donor heart under *ex situ* NMP is yet to be defined. Accumulated data will support an international consensus on the criteria.

In summary, this first case report shows the feasibility of IFBHT. This novel approach has potentially broad applications. From a clinical perspective, the technique may offer the opportunity to expand the donor pool to donors that are currently considered marginal. The approach may furthermore allow to reduce rates of primary non-function, acute rejection and CAV, thus improving transplant outcomes. From a research perspective, our approach is expected to significantly contribute to an improved understanding of the complex interaction between IRI and alloimmunity.

## Contributors

HXS is the conceptor of IFBHT; he organized the animal experiments and clinical trial, and critically revised the report. YSL, RJ, CYH, GZY and HXS designed the procedure and study protocol. YSL, RJ, JN and GZY analyzed the data and wrote the report. CL, MSY, SH, SYX and ZYL assisted design of NMP protocol and the preclinical preparation, as well as collected NMP parameters. LYQ, CYH, WTL and LHZ, assisted the design of the procedure, preclinical preparation and the operation. JN, XW, CYL, CXX, CXJ, GFQ, HWQ, and DYG assisted the design of the protocol and preclinical preparation. XGX and YMX analyzed the data and drafted the figures. BN and SGT critically revised the manuscript.

### Data sharing statement

Original data statistical analysis plan, informed consent form, and ethics committee approval are available and can be shared upon request. All available participant data will be de-identified. To access data, a request should be submitted to the corresponding author with a scientific proposal including objectives. The steering committee of this study will discuss and evaluate all requests and decide whether data sharing is appropriate. Data will only be shared after a data sharing agreement is fully executed

## Declaration of interests

The authors of this manuscript have no conflicts of interest to disclose as described by *The Lancet Regional Health – Western Pacific.*

## Appendix Supplementary materials

Image, application 1

Image, video 1

Image, video 2
