# Supplementary material for: Transplantation of a beating heart: A first in man
Source: Lancet Reg Health West Pac. 2022 Apr 12;23:100449. doi: 10.1016/j.lanwpc.2022.100449 (PMC9019404; doi:10.1016/j.lanwpc.2022.100449)
Supplement: Supplementary file 1 [file mmc1.docx]

**Supplementary Appendix**

**Supplement to: Shengli Yin, Jian Rong, Yinghua Chen, et al. Transplantation of a Beating Heart: A First in Man**

**SUPPLEMENTARY DOCUMENTS**

**Supplementary Table of Contents**

1. Supplemental Figure 1. Levels of myocardial injury markers pre- and post-transplantation – page 2

2. Supplemental Figure 2. The electrocardiogram (ECG) of the donor heart before donation and after transplantation– page 3


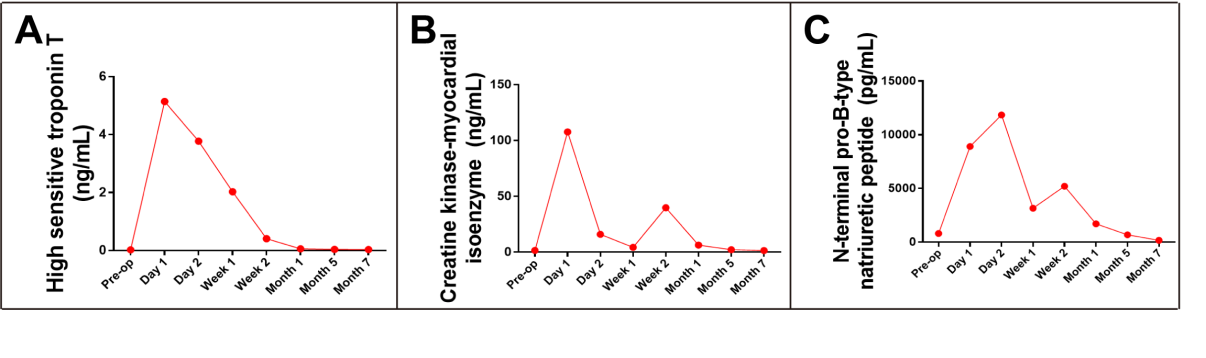


**Supplemental Figure 1. Levels of myocardial injury markers pre- and post-transplantation.** Levels of high sensitive troponin T (A), creatine kinase-myocardial isoenzyme (B), N-terminal pro-B-type natriuretic peptide (C) pre-transplantation and at Day 1, Day 2, Week 1, Week 2, Month 1, Month 5 and Month 7 post-transplantation.


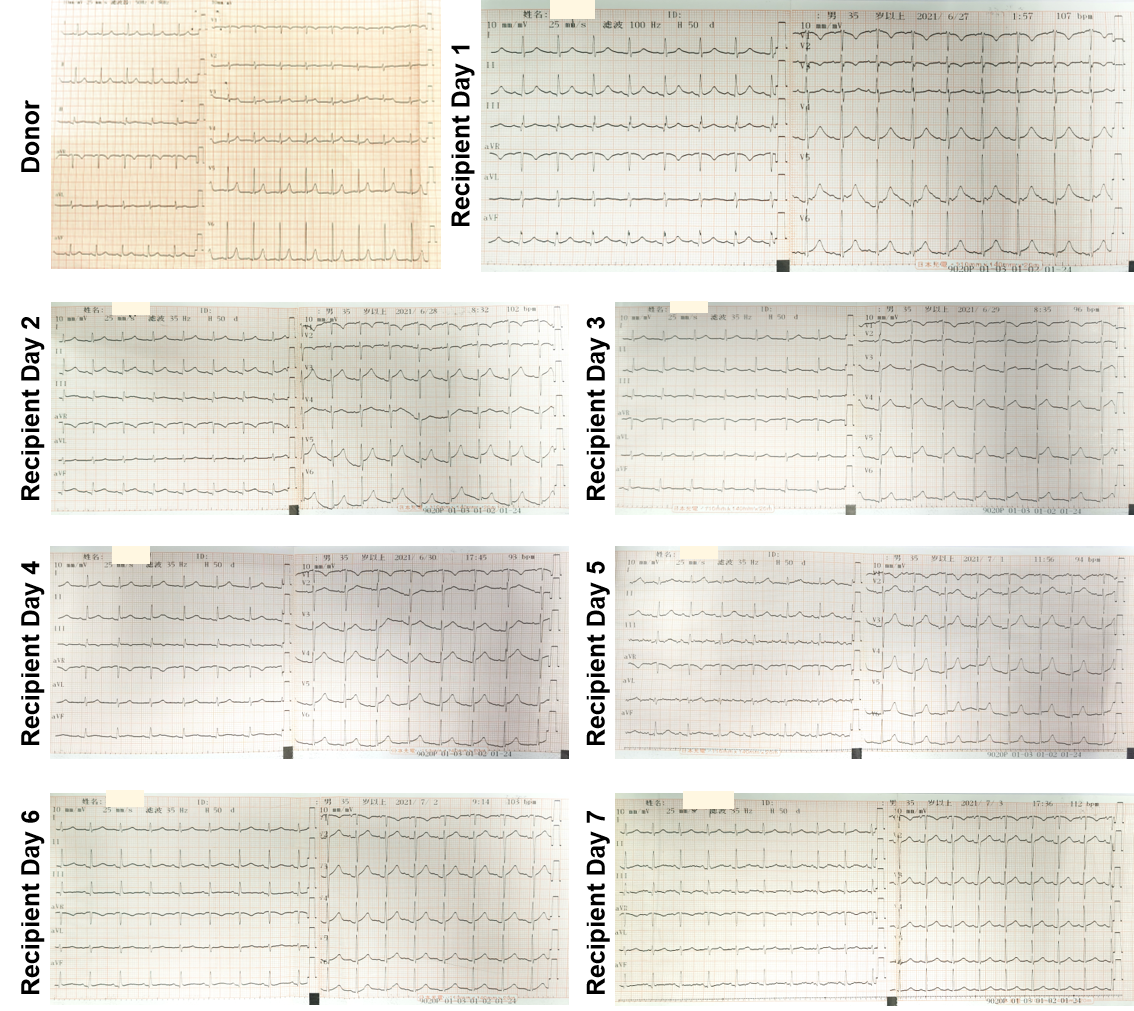


**Supplemental Figure 2. The electrocardiogram (ECG) of the donor heart before donation and after transplantation.**
